# Natural products in neurodegenerative diseases: recent advances and future outlook

**DOI:** 10.3389/fphar.2025.1529194

**Published:** 2025-03-19

**Authors:** L. Nahar, R. Charoensup, Kulyash Kalieva, E. Habibi, M. Guo, D. Wang, M. Kvasnica, A. Onder, S. D. Sarker

**Affiliations:** ^1^ Laboratory of Growth Regulators, Palacký University and Institute of Experimental Botany, The Czech Academy of Sciences, Olomouc, Czechia; ^2^ School of Integrative Medicine and Medicinal Plants Innovation Center of Mae Fah Luang University, Chiang Rai, Thailand; ^3^ Department of Chemistry and Mathematics, Al-Farabi Kazakh National University, Almaty, Kazakhstan; ^4^ Department of Pharmacognosy, Faculty of Pharmacy, Medicinal Plants Research Centre, Mazandaran University of Medical Sciences, Sari, Iran; ^5^ Laboratory of Advanced Theranostic Materials and Technology, Ningbo Institute of Materials Technology and Engineering, Chinese Academy of Sciences, Ningbo, China; ^6^ International Joint Laboratory of Medicinal Food Development and Health Products Creation, Biological Engineering Technology Innovation Center of Shandong Province, Heze Branch of Qilu University of Technology (Shandong Academy of Sciences), Heze, China; ^7^ Department of Pharmacognosy, Faculty of Pharmacy, Ankara University, Ankara, Türkiye; ^8^ Centre for Natural Products Discovery, School of Pharmacy and Biomolecular Sciences, Liverpool John Moores University, Liverpool, United Kingdom

**Keywords:** Alzheimer’s disease, Parkinson’s disease, Huntington’s disease, natural products, neurodegenerative diseases, neuroprotection, polyphenols, bioavailability

## Abstract

Neurodegenerative diseases such as Alzheimer’s, Parkinson’s, and Huntington’s are on the rise and pose significant challenges due to the lack of effective treatments. This review critically examines the neuroprotective effects of various natural products derived from plants, marine organisms, and fungi. Natural products have long been used in traditional medicine and are gaining attention in modern drug discovery for their unique properties. The review explains how these natural products can protect neurons by influencing the key biological pathways involved in neurodegeneration. It discusses mechanisms including antioxidant effects, anti-inflammatory actions, modulation of cellular signalling, and support for mitochondrial function. A systematic literature search was conducted to minimize bias and ensure rigorous study selection. Preclinical studies using animal models and cell cultures show that secondary metabolites like polyphenols, alkaloids, and terpenoids can significantly reduce neuronal damage. Some clinical trials have shown promising results. However, challenges such as bioavailability, standardization, and dosage must be addressed to translate these findings into clinical practice. The review also evaluates the potential synergy of combining natural products with conventional treatments, offering a complementary therapeutic approach. Natural products represent a promising avenue for developing innovative treatments for neurodegenerative diseases. The review highlights key research gaps and proposes future directions. Future studies should focus on overcoming existing challenges and refining these natural products to improve their efficacy and safety in clinical settings. The application of existing knowledge has the potential to significantly enhance the quality of life for individuals affected by neurodegenerative diseases.

## 1 Introduction

Neurodegenerative diseases are a growing global health problem. They damage the nervous system and affect cognitive functions. These diseases, including Alzheimer’s disease, Parkinson’s disease, and Huntington’s disease, are major public health challenges. They progressively worsen over time, severely affecting the quality of life for those who suffer from them (Wilson et al., 2023; Gadhave et al., 2024). Alzheimer’s disease primarily causes memory loss, cognitive decline, and behavioral changes, making it the most common form of dementia. Parkinson’s disease leads to motor issues like tremors, stiffness, and slow movement. Huntington’s disease is inherited and causes involuntary movements, psychiatric symptoms, and cognitive decline (Hardy and Selkoe, 2002; Dadgostar et al., 2022; Houldsworth, 2024). These diseases are becoming more common, putting pressure on healthcare systems worldwide (Wilson et al., 2023).

Despite progress in understanding their pathophysiology, current treatments are limited. They mostly relieve symptoms but do not halt or reverse the progression, highlighting the need for new and effective treatments (Kwon and Koh, 2020; Teleanu et al., 2022). Natural products have long been used in traditional medicine for their healing properties (Rahman et al., 2021; Tyler and Tyler, 2023). Plants, marine organisms, and fungi contain diverse secondary metabolites which play significant roles in many medicinal practices. These secondary metabolites possess unique chemical structures and biological activities, making them invaluable for drug discovery, particularly in neuroprotection. They offer antioxidant, anti-inflammatory, and neuroprotective effects, positioning them as promising alternatives to traditional drugs (Das et al., 2020; Teleanu et al., 2022). However, their pharmacological relevance requires critical evaluation, considering factors such as bioavailability, therapeutic efficacy, and mechanistic validation.

This review systematically examines the potential of natural products as therapeutic agents for neurodegenerative diseases. It differentiates between physiological and pharmacological effects, and their mechanisms of action. Based on preclinical and clinical evidence, the review highlights the challenges and opportunities of using natural products for neurodegenerative diseases. The aim of this review is to demonstrate how natural products can address the therapeutic needs of neurodegenerative diseases and encourage further research in this area. In summary, this review investigates natural products as a foundation for new drugs, offering hope for improved treatment options for neurodegenerative diseases.

## 2 Search strategy and study selection

A systematic and rigorous approach was employed to ensure a comprehensive and unbiased selection of relevant studies. Literature searches were conducted across multiple databases, including PubMed, Scopus, Web of Science, and Google Scholar. The search strategy utilized a combination of keywords such as “natural products,” “neurodegenerative diseases,” “Alzheimer’s disease,” “Parkinson’s disease,” “Huntington’s disease,” “neuroprotection,” and “pharmacological activities,” integrated with Boolean operators (AND, OR) to refine and optimize search results.

Studies were selected based on the following inclusion criteria: (1) investigation of the neuroprotective effects of natural products, (2) publication in peer-reviewed journals, (3) presentation of original experimental or clinical findings, and (4) availability in English. Exclusion criteria included non-English publications, review articles without original data, studies unrelated to neurodegenerative diseases or natural products, and research exclusively focused on synthetic compounds without direct links to natural sources. The selection process involved an initial screening of titles and abstracts to eliminate irrelevant studies, followed by a thorough full-text review to confirm eligibility based on the predefined inclusion and exclusion criteria.

## 3 Pathophysiology of neurodegenerative diseases

Neurodegenerative diseases (NDs) such as Alzheimer’s, Parkinson’s, and Huntington’s disease are marked by the gradual loss of neuronal structure and function. This leads to impairments in cognition, motor control, and behavioural changes. These disorders result from a complex interplay of genetic, environmental, and molecular factors (Wilson et al., 2023). An understanding of these mechanisms is crucial for developing effective therapeutic interventions. The pathophysiology of neurodegenerative diseases is highlighted in Table 1.

**TABLE 1:** The pathophysiology of neurodegenerative diseases.

Neurodegenerative diseases (ND)	Key features	Mechanisms	References
Alzheimer’s Disease (AD)	Aβ plaques; Tau tangles; Oxidative stress; Neuroinflammation; Mitochondrial dysfunction	Impaired synaptic function; Disrupted intracellular transport; Cellular damage; Increased cognitive decline; Impaired ATP production	Guo et al. (2013); Hampel et al. (2021); Chen et al. (2022); Teleanu et al. (2022); Zhang et al. (2023); Houldsworth (2024); Peggion et al. (2024)
Parkinson’s Disease (PD)	Lewy bodies; Oxidative stress; Neuroinflammation; Mitochondrial dysfunction; Impaired autophagy	Impaired synaptic transmission; Toxic free radicals; Accelerated neuronal loss; Disrupted energy metabolism; Hindered clearance of misfolded proteins	Spillantini et al. (1997); Trist et al. (2019); Yi et al. (2022); Peggion et al. (2024)
Huntington’s Disease (HD)	Mutant huntingtin protein; Oxidative stress; Excitotoxicity; Neuroinflammation	Disrupted transcription and transport; Neuronal damage; Disease progression	Guo et al. (2013); Jimenez-Sanchez et al. (2017); Fornari Laurindo et al. (2023); Jiang et al. (2023); Zhang et al. (2023); Tong et al. (2024)

### 3.1 Alzheimer’s disease

Alzheimer’s disease (AD) is the most prevalent form of dementia. It is characterized by cognitive decline, memory impairment, and behavioural changes. The key pathological features include amyloid-beta (Aβ) plaques and tau tangles in the brain (Hampel et al., 2021). Aβ plaques form from peptides cleaved from amyloid precursor protein (APP) by beta-secretase and gamma-secretase (Chen et al., 2022). These plaques impair synaptic function and neuronal communication. Tau tangles develop from hyperphosphorylated tau protein, which disrupts intracellular transport and neuronal stability (Houldsworth, 2024). Beyond plaques and tangles, oxidative stress, neuroinflammation, and mitochondrial dysfunction contribute significantly to AD progression (Zhang et al., 2023; Peggion et al., 2024). Oxidative stress, caused by reactive oxygen species (ROS) and reactive nitrogen species (RNS), damages cellular components, leading to neuronal injury and apoptosis (Houldsworth, 2024). Chronic neuroinflammation, driven by activated microglia and astrocytes, increases cognitive decline through pro-inflammatory cytokines (Guo et al., 2013). Mitochondrial dysfunction impairs ATP production and increases ROS levels, further promoting neuronal degeneration (Teleanu et al., 2022; Peggion et al., 2024).

### 3.2 Parkinson’s disease

Parkinson’s disease (PD) is primarily a movement disorder. It results from the loss of dopaminergic neurons in the substantia nigra, a region crucial for movement regulation. The formation of Lewy bodies, which contain aggregates of the alpha-synuclein protein, is the main pathological hallmark of PD (Spillantini et al., 1997). These aggregates impair synaptic transmission and disrupt cellular homeostasis (Yi et al., 2022). Oxidative stress plays a significant role in PD, particularly due to the high iron and dopamine levels in the substantia nigra, which lead to the production of toxic free radicals. Neuroinflammation, triggered by activated microglia, accelerates neuronal loss (Trist et al., 2019; Yi et al., 2022). Mitochondrial dysfunction and impaired autophagy disrupt energy metabolism and the clearance of misfolded proteins, increasing damage (Peggion et al., 2024).

### 3.3 Huntington’s disease

Huntington’s disease (HD) is a genetic disorder characterized by movement abnormalities, psychiatric symptoms, and cognitive decline (Jimenez-Sanchez et al., 2017). It is caused by an expanded CAG repeat in the huntingtin gene (HTT), leading to a mutant huntingtin protein (mHTT) (Tong et al., 2024). This mutation results in protein misfolding and aggregation, disrupting essential cellular processes such as transcription, mitochondrial function, and intracellular transport. These disruptions ultimately cause neuronal dysfunction and death (Jiang et al., 2023). In HD, oxidative stress and excitotoxicity from excessive glutamate release play critical roles (Guo et al., 2013). Overall, an increased level of oxidative stress, excitotoxicity, and neuroinflammation collectively contribute to neuronal damage and the progression of neurodegenerative diseases (Zhang et al., 2023; Fornari Laurindo et al., 2023).

## 4 Common mechanisms in neurodegenerative diseases and

Neurodegenerative diseases such as Alzheimer’s, Parkinson’s, and Huntington’s share several common pathological mechanisms that contribute to disease progression. Understanding these overlapping pathways not only highlights the complexities of disease progression but also identifies potential targets for therapeutic interventions. The pathophysiology of neurodegenerative diseases revolves around several interconnected mechanisms such as protein aggregation, oxidative stress, mitochondrial dysfunction, neuroinflammation, and excitotoxicity.

A hallmark of neurodegenerative diseases is the misfolding and aggregation of specific proteins. In AD, Aβ peptides and hyperphosphorylated tau form toxic aggregates that impair synaptic function and disrupt neuronal stability (Hampel et al., 2021; Zhang et al., 2023). In PD, alpha-synuclein accumulates in Lewy bodies, leading to synaptic dysfunction and neuronal death (Spillantini et al., 1997; Yi et al., 2022). In HD, mutant huntingtin protein aggregates interfere with essential cellular processes, exacerbating neuronal degeneration (Jiang et al., 2023). These aggregated proteins disrupt proteostasis and impair cellular functions, ultimately leading to neuronal loss. The pathophysiology of neurodegenerative diseases which is outlined in Table 2.

**TABLE 2:** Common mechanisms in neurodegenerative diseases.

Pathways	Description	References
Protein aggregation	Misfolding and aggregation of specific proteins (Aβ in AD; alpha-synuclein in PD; mutant huntingtin in HD)	Spillantini et al. (1997); Hampel et al. (2021); Zhang et al. (2023); Yi et al. (2022); Jiang et al. (2023)
Oxidative stress	ROS and RNS induce lipid peroxidation, DNA damage, and protein oxidation	Guo et al. (2013); Teleanu et al. (2022); Houldsworth (2024); Zhang et al. (2023); Peggion et al. (2024)
Mitochondrial dysfunction	Impaired ATP production and increased free radical generation	Trist et al. (2019); Peggion et al. (2024)
Neuroinflammation	Activated microglia and astrocytes release pro-inflammatory cytokines	Kwon and Koh (2020); Teleanu et al. (2022); Fornari Laurindo et al. (2023)
excitotoxicity	Excessive glutamate stimulation leads to calcium influx and cell death pathways	Jimenez-Sanchez et al. (2017); Zhang et al. (2023)
Dysfunction of protein degradation systems	Impaired UPS and autophagy lead to toxic accumulation of misfolded proteins	Dadgostar et al. (2022); Gadhave et al. (2024)

### 4.1 Antioxidant effects

Oxidative stress plays a central role in all three diseases (Teleanu et al., 2022). ROS and RNS induce lipid peroxidation, DNA damage, and protein oxidation, promoting neuronal death (Guo et al., 2013; Houldsworth, 2024). Natural compounds such as polyphenols, flavonoids, and carotenoids possess strong antioxidant properties. They can neutralize ROS, reduce oxidative damage to neurons, and enhance the activity of endogenous antioxidant enzymes (Zhang et al., 2023; Peggion et al., 2024).

### 4.2 Support for mitochondrial function

Mitochondrial dysfunction exacerbates oxidative damage by impairing ATP production and increasing free radical generation (Peggion et al., 2024). The dysfunctional mitochondria are unable to fulfil the elevated energy requirements of neurons, resulting in metabolic failure and triggering apoptotic cell death (Trist et al., 2019). Natural compounds like coenzyme Q10, quercetin, and berberine support mitochondrial function by enhancing mitochondrial biogenesis, improving electron transport chain efficiency, and reducing mitochondrial oxidative stress.

### 4.3 Anti-inflammatory actions

Chronic neuroinflammation is a common feature of neurodegenerative diseases (Kwon and Koh, 2020). In all three disorders, activated microglia and astrocytes release pro-inflammatory cytokines such as tumor necrosis factor-alpha (TNF-α) and interleukins, contributing to neuronal damage (Teleanu et al., 2022; Fornari Laurindo et al., 2023). Natural compounds like curcumin, resveratrol, and omega-3 fatty acids exhibit anti-inflammatory effects by inhibiting pro-inflammatory cytokines and signaling pathways, reducing neuroinflammation and protecting neuronal integrity.

### 4.4 Modulation of cellular signaling

Excitotoxicity, primarily caused by excessive glutamate stimulation, is particularly relevant in HD but also contributes to AD and PD pathology (Jimenez-Sanchez et al., 2017). The overactivation of glutamate receptors leads to calcium influx, mitochondrial overload, and activation of cell death pathways (Zhang et al., 2023). Natural compounds can modulate various cellular signaling pathways involved in neurodegeneration, such as the PI3K/Akt, MAPK, and Nrf2/ARE pathways, promoting neuronal survival and function.

### 4.5 Enhancement of protein degradation systems

A common characteristic of neurodegenerative diseases is the dysfunction of protein degradation systems, particularly the ubiquitin-proteasome system (UPS) and autophagy (Dadgostar et al., 2022). This dysfunction leads to the inability to clear misfolded proteins, resulting in toxic accumulation that further promotes neuronal dysfunction. Natural compounds can enhance autophagy and proteasomal activity, improving the clearance of harmful aggregates (Gadhave et al., 2024).

## 5 Neuroprotective effects and sources of natural products

Natural products sourced from plants, fungi, and marine organisms are increasingly recognized for their potential to mitigate neurodegenerative disease progression through diverse neuroprotective mechanisms (Jivishov et al., 2020). Plant-derived natural products have been extensively studied for their therapeutic potential in treating NDs such as AD, PD, and HD (Rahman et al., 2021; Tyler and Tyler, 2023). Plants biosynthesise a diverse array of secondary metabolites, including polyphenols, alkaloids, and terpenoids, long used in traditional medicine to support brain health. The neuroprotective effects and sources of natural products are depicted in Table 3.

**TABLE 3:** Neuroprotective effects and sources of natural products.

Natural product	Source	Neuroprotective effects	References
Resveratrol	Grapes, red wine	Reduces oxidative stress; Modulates inflammatory pathways	Albani et al. (2010); Dos Santos et al. (2024)
Curcumin	Turmeric	Inhibits Aβ aggregation; Reduces tau tangles; Improves cognitive function	Sarker and Nahar (2007); Esmaealzadeh et al. (2024)
EGCG	Green tea	Scavenges free radicals; Reduces inflammation	Fernandes et al. (2021)
Huperzine A	Chinese club moss	Increases acetylcholine levels; Improves memory and cognitive functions	Yang et al. (2019); Syed et al. (2021)
Berberine	Berberis species	Anti-inflammatory; Modulates glucose metabolism	Dadgostar et al. (2022)
Ginkgolides	*Ginkgo biloba*	Improves blood flow; Antioxidant properties	Singh et al. (2019)
Cannabidiol (CBD)	*Cannabis sativa*	Reduces neuroinflammation; Promotes neurogenesis	Zhang et al. (2023)
Omega-3 fatty scids	Fish oils	Supports neuronal membrane fluidity; Anti-inflammatory effects	Chitre et al. (2019)
Astaxanthin	Shellfish, shrimp, trout, salmon, microalgae	Antioxidant; Anti-inflammatory	Tanvir et al. (2022)
Reishi	*Ganoderma lucidum*	Reduces oxidative damage; Stimulates NGF synthesis	Saitsu et al. (2019); Chen et al. (2022)
Lion’s Mane	*Hericium erinaceus*	Promotes neurogenesis	Saitsu et al., 2019; Chen et al., 2022
Phlorotannins	Brown algae	Antioxidant properties; Reduces oxidative stress and neuroinflammation	Martins et al. (2020); Catanesi et al. (2021); Silva et al. (2023)

**FIGURE 1:**
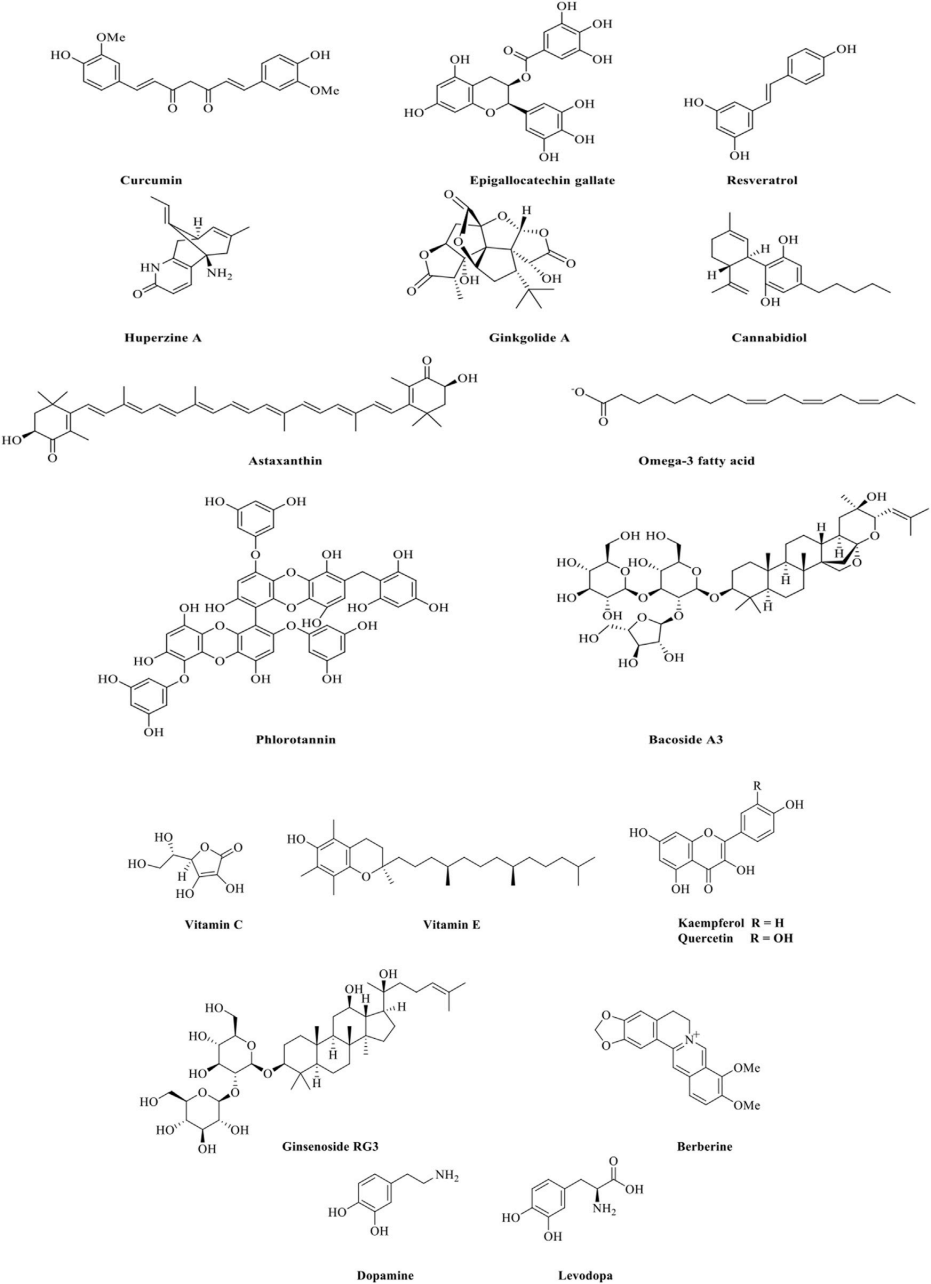
Structures of the selected compounds which are effective in neurodegenerative diseases.

Polyphenols are abundant in fruits, vegetables, tea, and wine. They possess potent antioxidant and anti-inflammatory properties, making them effective in reducing oxidative stress and neuroinflammation, which are key contributors to neurodegeneration. Resveratrol (Figure 1), found in grapes and red wine, supports neuronal health by reducing oxidative stress and modulating inflammatory pathways (Dos Santos et al., 2024). *In vitro* studies demonstrate that resveratrol (5–25 µM) mitigates oxidative damage by decreasing reactive oxygen species (ROS) accumulation in rat hippocampal neurons exposed to nitric oxide free radical donors (Albani et al., 2010).

Curcumin (Figure 1), derived from turmeric (*Curcuma longa* L., Fam: Zingiberaceae), has been extensively studied for its neuroprotective properties (Sarker and Nahar, 2007). It inhibits Ab aggregation, reduces tau tangles, and improves cognitive function in AD models (Esmaealzadeh et al., 2024). Another polyphenol, epigallocatechin gallate (EGCG) (Figure 1), from green tea (*Camellia sinensis* (L.) Kuntze., Fam: Theaceae), protects neurons by scavenging free radicals and reducing inflammation (Fernandes et al., 2021).

Alkaloids are nitrogenous secondary metabolites that have at least one nitrogen atom forming a ring, and they are biosynthesized by plants and microbes (Nahar and Sarker, 2019). They are known for their ability to modulate neurotransmitter systems and enhance cognitive function. Huperzine A (Figure 1), extracted from the Chinese club moss (*Huperzia serrata* (Thunb.) Trevis, Fam: Lycopodiaceae), acts as an acetylcholinesterase inhibitor, increasing acetylcholine levels and improving memory and cognitive functions in Alzheimer’s disease (Yang et al., 2019; Syed et al., 2021). Berberine, an alkaloid from *Berberis* species, shows anti-inflammatory properties and the ability to modulate glucose metabolism, potentially aiding in both AD and PD models (Dadgostar et al., 2022).

Terpenoids are abundant in essential oils and are responsible for the aromatic properties of many plants. They possess anti-inflammatory and antioxidant properties that protect neuronal integrity. Ginkgolides (Figure 1), from *Ginkgo biloba* L. (Fam: Ginkgoaceae), improve blood flow and exhibit antioxidant properties, offering protective effects in conditions like dementia and cognitive impairment (Singh et al., 2019).

Cannabidiol (CBD) (Figure 1), a non-psychoactive secondary metabolite found in *Cannabis sativa* (Fam: Cannabaceae) shows promise in reducing neuroinflammation and oxidative stress while promoting neurogenesis (Zhang et al., 2023). Omega-3 fatty acids, prevalent in fish oils, support neuronal membrane fluidity and exert anti-inflammatory effects, helping manage diseases like AD and PD (Chitre et al., 2019). Astaxanthin (Figure 1), a carotenoid found in shellfish, shrimp, trout, salmon, and microalgae, contains both ketone and hydroxyl groups. It is recognized as a powerful antioxidant with neuroprotective capabilities, primarily through the activation of the antioxidant network, including catalase and superoxide dismutase (SOD), which help combat oxidative stress and inflammation. Additionally, astaxanthin acts as a strong anti-inflammatory agent in the nervous system by inhibiting inflammatory pathways and providing protection to neurons in various neurodegenerative diseases, including AD and PD (Tanvir et al., 2022).

Fungi produce unique secondary metabolites that also exhibit neuroprotective effects. Reishi (*Ganoderma lucidum* Karst, Fam: Ganodermataceae) and lion’s mane (*Hericium erinaceus* (Bull.) Persoon, Fam: Hericiaceae) mushrooms contain polysaccharides and various secondary metabolites that stimulate nerve growth factor (NGF) synthesis and support neuron health. These triterpenes extracted from *G. lucidum* have been shown to reduce oxidative damage, while secondary metabolites from lion’s mane mushrooms can promote neurogenesis, offering potential benefits for AD and PD (Saitsu et al., 2019; Chen et al., 2022).

Marine environments offer a rich source of unique natural products with the potential to treat NDs like AD and PD (Catanesi et al., 2021). Marine organisms, including algae and sponges, produce omega-3 fatty acids and phlorotannins (Figure 1), shaped by their adaptation to harsh environmental conditions. Omega-3 fatty acids, especially those found in fish oils, support neuronal membrane fluidity and exhibit anti-inflammatory effects, benefiting both AD and PD models (Chitre et al., 2019; Fakhri et al., 2021).

Phlorotannins (Figure 1) extracted from brown algae have potent antioxidant properties and show potential for reducing oxidative stress and neuroinflammation, which are central to neurodegeneration (Silva et al., 2023). These marine natural products showed multifaceted activities highlighting the therapeutic potential of natural products, providing a foundation for further exploration in ND treatments (Martins et al., 2020; Catanesi et al., 2021).

## 6 Mechanisms of action of natural products

Natural products exert neuroprotective effects through various mechanisms that are crucial for combating NDs (Tyler and Tyler, 2023). They also inhibit protein aggregation, a hallmark of neurodegenerative diseases. These mechanisms include reducing oxidative stress, exerting anti-inflammatory effects, inhibiting protein aggregation, and other neuroprotective strategies (Das et al., 2020). Several studies showed that plant-derived secondary metabolites can protect mitochondrial function by enhancing biogenesis, reducing oxidative stress, and preventing membrane depolarization. Studies indicate that EGCG (Figure 1) has protective effects on mitochondria in AD and HD models by activating AMP-activated protein kinase (AMPK) and reducing mitochondrial ROS production (Fernandes et al., 2021; Gonçalves et al., 2021).

Several NDs involve the activation of apoptotic pathways leading to neuronal death. In addition, plant-derived natural products have been shown to inhibit these pathways, preserving neuronal integrity. *Bacopa monnieri* (L.) Wettst (Fam: Plantaginaceae), known for its secondary metabolites like bacosides (Figure 1), has been found to inhibit apoptosis by regulating Bcl-2 family proteins and preventing mitochondrial cytochrome c release in AD models (Abdul Manap et al., 2019). The mechanisms of action of natural products in neurodegenerative diseases is shown in Table 4.

**TABLE 4:** Neuroprotective effects and sources of natural products.

Mechanism	Description	References
Oxidative Stress Reduction	Antioxidants neutralize ROS; Enhance activity of antioxidant enzymes (SOD, catalase)	Guo et al. (2013); Solanki et al. (2015); Jin et al. (2023); Dos Santos et al. (2024); Esmaealzadeh et al. (2024); Jiang et al. (2023)
Anti-inflammatory Effects	Modulate key pathways; Lower harmful cytokines (TNF-α, IL-6); Inhibit transcription factors (NF-κB)	Burgos et al. (2015); Kwon and Koh (2020); Rahman et al. (2021); Fakhri et al. (2021); Esmaealzadeh et al. (2024); Zhang et al. (2023); Teleanu et al. (2022); Jin et al. (2023)
Inhibition of Protein Aggregation	Prevent toxic oligomer and fibril formation; Enhance autophagic processes	Spillantini et al. (1997); Rezai-Zadeh et al. (2005); Javed et al. (2018); Hampel et al. (2021); Arbo et al. (2020); Tian et al. (2023)
Modulation of Neurotransmitter Systems and Neurogenesis	Enhance acetylcholine levels; Replenish dopamine levels; Improve mitochondrial function	Katzenschlager et al. (2004); Yang et al. (2019); Syed et al. (2021)

### 6.1 Oxidative stress reduction

Oxidative stress is a significant contributor to neuronal damage in neurodegenerative diseases (Houldsworth, 2024). Natural products like flavonoids, polyphenols, and vitamins, such as vitamin C and E (Figure 1), act as antioxidants, neutralizing reactive oxygen species (ROS) and reducing oxidative stress (Guo et al., 2013; Solanki et al., 2015; Jin et al., 2023). Polyphenols like curcumin (Figure 1), and resveratrol (Figure 1) have shown the ability to lower oxidative stress in neuronal cells (Dos Santos et al., 2024; Esmaealzadeh et al., 2024). These natural products enhance the activity of antioxidant enzymes, such as superoxide dismutase (SOD) and catalase, thereby protecting neurons from oxidative damage and preserving cognitive function (Jiang et al., 2023).

### 6.2 Anti-inflammatory effects

Chronic inflammation in the brain, often triggered by activated microglia, plays a key role in neurodegeneration (Kwon and Koh, 2020). Natural products help reduce inflammation by modulating key pathways (Rahman et al., 2021). Polyphenols such as curcumin (Figure 1), epigallocatechin gallate (EGCG) (Figure 1) and resveratrol (Figure 1), exhibit anti-inflammatory effects by lowering harmful cytokines like TNF-α and IL-6 and inhibiting transcription factors like NF-κB (Fakhri et al., 2021; Esmaealzadeh et al., 2024). Although, the biological effects of EGCG are contingent on concentration levels. The plasma concentrations ≤10 μM elicit antioxidant, anti-inflammatory, and insulin-sensitizing effects (Alam et al., 2022).

Resveratrol (Figure 1) has demonstrated a decrease in neuroinflammation in AD models by preventing microglial activation., further safeguarding cognitive function (Arbo et al., 2020). Resveratrol effectively inhibited LPS-induced production of proinflammatory factors (NO, TNFα, IL-1β) in neuron-glia cultures, offering significant neuroprotection to dopamine neurons in Parkinson’s disease. It reduced microglia-derived TNFα at 3 h, and NO and IL-1β at 24 h, across various concentrations and time points. Moreover, resveratrol treatment significantly inhibited the LPS-induced production of these proinflammatory factors in the supernatant of neuron-glia cultures (TNFα, NO and IL-1β). Post hoc tests indicated that, at a concentration of 30 μM, resveratrol significantly reduced the secretion of TNFα, IL-1β and NO. At a higher concentration of 60 μM, resveratrol markedly suppressed the production of TNFα, IL-1β and NO (Zhang et al., 2023). Furthermore, resveratrol showed inhibitory effects on LPS-induced activation of MAPKs (ERK1/2, p38, and JNK) in microglia-enriched cultures. Pretreatment with resveratrol significantly reduced LPS-induced phosphorylation of ERK1/2, p38 and JNK confirmed by *post hoc* tests for each MAPK (Zhang et al., 2023).

Flavonoids like quercetin and kaempferol (Figure 1) also inhibit the activation of microglia and astrocytes, reducing the production of pro-inflammatory cytokines (Teleanu et al., 2022; Jin et al., 2023). These actions alleviate brain inflammation and may slow the progression of neurodegenerative diseases (Zhang et al., 2023). Curcumin (Figure 1) effectively reduces neuroinflammation by inhibiting the NF-κB pathway, which is pivotal in inflammatory responses in the brain (Esmaealzadeh et al., 2024). Ginsenosides (Figure 1) from *Panax ginseng* C.A. Mey. also reduce inflammation in PD models by downregulating pro-inflammatory cytokines and reducing microglial activation (Burgos et al., 2015).

### 6.3 Inhibition of protein aggregation

Protein misfolding and aggregation, such as amyloid-beta (Ab) plaques in AD and alpha-synuclein aggregates in PD, are central to the pathology of many neurodegenerative conditions (Spillantini et al., 1997). Bioactive secondary metabolites like EGCG (Figure 1) from green tea inhibit protein aggregation, preventing toxic oligomer and fibril formation (Rezai-Zadeh et al., 2005).

*In vitro* investigations have shown that Aβ deposition significantly decreases following intraperitoneal injection of EGCG at a dose of 20 mg/kg or oral administration of EGCG at 50 mg/kg in drinking water. In addition, a substantial reduction in Aβ deposition was observed in the frontal cortex (60%) and hippocampus (52%) following oral administration of EGCG at a dose of 20 mg/kg/day for 3 months in an AD mouse model. *In vitro* studies utilised SH-SY5Y neuronal cells to evaluate neuroprotection, while *in vivo* studies employed transgenic mouse models of AD, specifically the APP/PS1 model. These models are well-recognized for their relevance in investigating the mechanisms of AD (Rezai-Zadeh et al., 2005; Li et al., 2006).

Curcumin (Figure 1) binds to Aβ, inhibiting its aggregation and promoting toxic protein clearance (Hampel et al., 2021). Resveratrol (Figure 1) has shown the ability to reduce Aβ aggregation, enhance autophagic processes to clear aggregated proteins from neurons and improve cognitive function in preclinical models (Arbo et al., 2020). Similarly, berberine (Figure 1), an alkaloid found in *Berberis* species, shows promise in inhibiting alpha-synuclein aggregation in PD models, preventing the formation of Lewy bodies (Javed et al., 2018; Tian et al., 2023).

### 6.4 Modulation of neurotransmitter systems and neurogenesis

**FIGURE 2:**
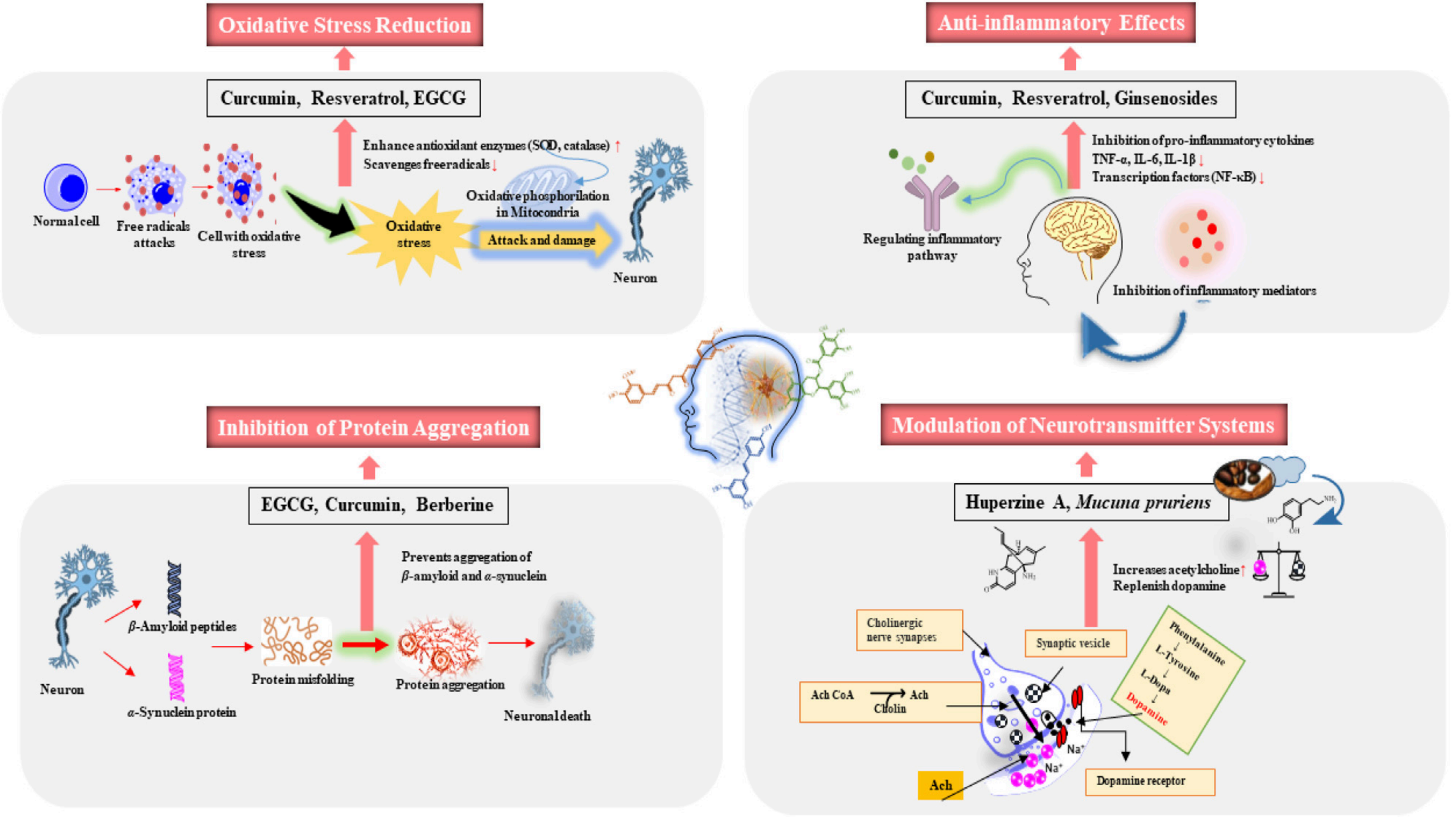
The mechanisms of selected phytochemicals in neurodegenerative diseases.

Beyond oxidative stress reduction, anti-inflammatory effects, and inhibition of protein aggregation, natural products also enhance neurogenesis, improve mitochondrial function, and modulate neurotransmitter systems. Many plant-derived secondary metabolites can modulate neurotransmitter systems, particularly cholinergic and dopaminergic pathways, which are often impaired in NDs (Syed et al., 2021). Huperzine A (Figure 1) inhibits acetylcholinesterase, enhancing acetylcholine levels critical for cognitive functions (Yang et al., 2019). In PD, plants like *Mucuna pruriens* (L.) DC., (Fam: Fabaceae) which contains levodopa (L-DOPA) (Figure 1), is used to replenish dopamine (Figure 1) levels, addressing motor deficits characteristic of the disease (Katzenschlager et al., 2004). A diagram contains mechanisms underlying the neuroprotective effects of natural compounds presented in (Figure 2).

## 7 Preclinical studies in animal models

Preclinical studies using animal models provide critical insights into the therapeutic potential of natural products for neurodegenerative diseases, allowing researchers to assess efficacy, safety, and mechanisms of action in controlled environments. Polyphenols such as curcumin, resveratrol, and EGCG have demonstrated notable effects in animal models of AD, reducing Aβ plaques, oxidative stress, and neuroinflammation while improving cognitive function. Polyphenols such as curcumin, resveratrol, and EGCG (Figure 1) have demonstrated notable effects in animal models of AD, reducing Aβ plaques, oxidative stress, and neuroinflammation while improving cognitive function (Solanki et al., 2015; Gonçalves et al., 2021; Hampel et al., 2021; Esmaealzadeh et al., 2024). These studies allow researchers to assess efficacy, safety, and mechanisms of action in controlled environments. The effects of various natural products in animal models of neurodegenerative diseases are depicted below in Table 5.

**TABLE 5:** The effects of various natural products in animal models of neurodegenerative diseases.

Natural product	Animal model	Effects
Curcumin	AD models (rats)	Reduces oxidative stress, neuroinflammation, neurodegeneration; Improves cognitive function
Resveratrol	PD models (rats)	Reduces oxidative stress; Supports dopaminergic neuron survival; Activates sirtuin pathways
Berberine	PD models	Inhibits alpha-synuclein aggregation; Prevents Lewy body formation
Ginsenosides	AD and PD models (rats, mice)	Promotes neurogenesis; Modulates neuroinflammatory responses; Improves memory
Omega-3 Fatty Acids	AD and PD models	Reduces neuroinflammation; Maintains synaptic function; Decreases Aβ plaque formation
Phlorotannins	AD and PD models	Reduces oxidative damage; Inhibits neurotoxic protein aggregation
Polysaccharides	AD models	Modulates neuroinflammatory responses; Supports neurogenesis

Research demonstrated the neuroprotective effects of curcumin against LPS-induced oxidative stress, neuroinflammation, neurodegeneration, and memory deficits in the hippocampus of adult rats. These effects are mediated through the regulation of the JNK/NF-κB/Akt signaling pathway. In that study, rats were administered LPS (250 μg/kg) intraperitoneally for 7 days. They were then administered curcumin (300 mg/kg) for 14 days. After the complete treatment, assessments were conducted to evaluate reactive oxygen species, lipid peroxidation, and protein expression through western blotting. In addition, HT-22 neuronal and BV2 microglial cells were treated with LPS (1 μg/mL), curcumin (100 μg/mL), and the JNK inhibitor SP600125 (20 μM) to confirm the role of the pathway. It was shown that curcumin could effectively reduce LPS-induced oxidative stress and neuroinflammation. It mitigated neuronal cell death, and improved cognitive performance in memory tasks. In that study, curcumin was tested at doses ranging from 5 to 25 µM in *in vitro* models. *In vivo* studies utilized a dose of 300 mg/kg to assess neuroprotection. The minimal concentration required to reduce oxidative stress in hippocampal cells was established at 5 µM (Khan et al., 2019).

In animal models of PD, resveratrol and berberine have shown neuroprotective effects. They reduce oxidative stress and support the survival of the dopaminergic neurons, which is essential for improving motor function. Resveratrol has the ability to activate sirtuin pathways and contribute to its neuroprotective profile. This makes it particularly relevant in PD animal studies, where oxidative damage is a key pathological factor (Azargoonjahromi and Abutalebian, 2024; Dos Santos et al., 2024).

Ginsenosides (Figure 1), the active components of ginseng, have shown neuroprotective effects in PD and AD models by promoting neurogenesis and modulating neuroinflammatory responses, which protect neurons from oxidative and inflammatory stress (Burgos et al., 2015). In the behavioral assessments, male Sprague-Dawley rats treated with *Panax ginseng* extract at the doses of 1 g/kg, 0.5 g/kg, and 0.25 g/kg exhibited significant improvements. They showed shortened escape latency, increased crossing times, reduced errors, and prolonged latency in rats with AGE-induced AD. Moreover, ginseng treatment decreased malondialdehyde levels, increased glutathione content, and enhanced SOD activity in the hippocampus (Tan et al., 2015). The beneficial effects of ginsenosides were evident in the prevention of memory loss in aged SAMP8 mice. The optimal dose of ginsenosides was found to be 100 or 200 mg/kg per day for 7 months. This treatment resulted in marked reductions in Aβ levels in the hippocampus and significant increases in antioxidative enzyme levels in serum (Zhao et al., 2009).

Marine-derived natural products, including omega-3 fatty acids and phlorotannins (Figure 1) have demonstrated significant neuroprotective potential in preclinical models of neurodegenerative diseases. Omega-3 fatty acids (Figure 1), abundant in fish oils, have shown remarkable efficacy in reducing neuroinflammation and maintaining synaptic function, particularly in models of AD and PD. These fatty acids support neuronal membrane fluidity and decrease inflammatory responses, thus protecting neurons from the damaging effects of oxidative stress and inflammation. In animal models of AD, omega-3 supplementation has been associated with reduced Aβ plaque formation and improved cognitive function. This indicates their potential as adjunctive agents in neurodegenerative therapy (Chitre et al., 2019; Rivai and Umar, 2023).

Phlorotannins (Figure 1), a unique class of polyphenolics derived from brown seaweed, exhibit strong neuroprotective effects. These polyphenols are known for their potent antioxidant properties. They help neutralize ROS and prevent oxidative stress, which is a central factor in the pathology of AD, PD, and HD. Phlorotannins have shown promise in animal models for their multitarget actions, as they not only reduce oxidative damage but also inhibit the aggregation of neurotoxic proteins like Ab in AD and alpha-synuclein in PD. This dual action against oxidative stress and protein misfolding highlights phlorotannins as promising candidates for further investigation in neurodegenerative disease models (Fakhri et al., 2021; Silva et al., 2023).

In addition, polysaccharides from marine sources, such as sulphated glycosaminoglycans, have been studied for their ability to modulate neuroinflammatory responses and support neurogenesis. These polysaccharides have shown benefits in preclinical models by inhibiting pro-inflammatory cytokines. This inhibition reduces chronic inflammation, which is detrimental in neurodegenerative conditions. Studies in AD models suggest that these natural products can protect neurons by promoting anti-inflammatory pathways. Consequently, this enhances cognitive performance and delays neurodegeneration (Lomartire and Gonçalves, 2023).

The diverse array of secondary metabolites from marine sources highlights the therapeutic potential of these natural products in treating neurodegenerative diseases (Fakhri et al., 2021; Silva et al., 2023). The promising results from animal studies form a foundation for future clinical research aimed at translating these findings into effective human therapies (Tan et al., 2015; Rivai & Umar, 2023). By addressing multiple pathogenic pathways, such as oxidative stress, protein aggregation, and neuroinflammation, marine natural products may offer comprehensive neuroprotection (Chitre et al., 2019; Gadhave et al., 2024). This highlights the potential of marine natural products as a valuable resource in neurodegenerative disease management. Selected *in vitro* models, such as lipopolysaccharide (LPS)-induced microglial activation, are valuable for studying neuroinflammatory responses (Lomartire and Gonçalves, 2023). However, these models do not replicate the complexities of *in vivo* conditions. Therefore, further animal studies are necessary to validate these findings.

## 8 Clinical studies of natural products

Clinical trials have demonstrated encouraging, though variable, outcomes for several natural products in treating neurodegenerative diseases. Curcumin has been tested in randomized, double-blind, placebo-controlled trials, revealing a reduction in Aβ plaques and enhanced cognitive function among patients with mild to moderate AD. This reduction in Aβ burden has been attributed to curcumin’s anti-inflammatory and antioxidant effects, offering a promising adjunct to traditional AD treatments (Azargoonjahromi and Abutalebian, 2024; Esmaealzadeh et al., 2024). A Summary of clinical studies of natural compounds in neurodegenerative diseases is presented in Table 6.

**TABLE 6:** The effects of various natural products in animal models.

Natural product	Animal model	Clinical outcomes	References
Curcumin	AD models (rats)	Reduction in Aβ plaques, enhanced cognitive function	Azargoonjahromi and Abutalebian (2024); Esmaealzadeh et al. (2024)
*Ginkgo biloba* extract	AD models (rats)	Improved cognitive function, delayed disease progression	Singh et al. (2019); Dos Santos et al. (2024)
Resveratrol	PD models (rats)	Improved motor functions, reduced oxidative biomarkers	Azargoonjahromi and Abutalebian (2024)
Ginseng extracts	PD models (rats, mice)	Improved motor function, decreased neuroinflammatory markers	Kim et al. (2018); Burgos et al. (2015)
EGCG	HD models (rats)	Improved motor and cognitive functions	Fernandes et al. (2021); Jiang et al. (2023)

*Ginkgo biloba* extract, widely used in Asia and Europe for cognitive enhancement, has also been explored in multiple clinical studies. These studies suggest that ginkgolides can improve cognitive function, particularly in the early stages of AD, and may delay disease progression when used as a supplement. The neuroprotective properties of *G. biloba* extract are believed to arise from their antioxidant effects and capacity to enhance cerebral blood flow, potentially protecting neurons from oxidative stress-related damage (Singh et al., 2019; Dos Santos et al., 2024).

In PD models, resveratrol has been studied for its potential to mitigate motor symptoms and oxidative stress. As an antioxidant and sirtuin activator, resveratrol showed promise in protecting dopaminergic neurons and reducing oxidative stress. Clinical studies indicate improvements in motor functions and reductions in oxidative biomarkers, making resveratrol a candidate for integrative PD therapy (Azargoonjahromi and Abutalebian, 2024). Ginseng extracts, containing active ginsenosides (Figure 1), have been investigated in PD patients. Research showed improvements in motor function and a decrease in neuroinflammatory markers. The effects of ginseng on PD are linked to its ability to reduce oxidative stress and modulate immune responses, making it a potentially valuable adjunctive therapy in managing PD symptoms (Kim et al., 2018; Burgos et al., 2015).

The polyphenol epigallocatechin gallate (found in green tea) has also been tested in HD models and patients. It showed potential to improve both motor and cognitive functions. As HD is marked by oxidative stress and excitotoxicity, the antioxidant properties of EGCG play a critical role in reducing these pathologies. Clinical trials have reported improvements in motor coordination and cognitive parameters, supporting EGCG as a multi-target agent with a strong neuroprotective profile (Fernandes et al., 2021; Jiang et al., 2023). Despite these promising findings, the clinical application of natural products faces significant challenges, primarily due to issues with bioavailability, variability in product composition, and inconsistent therapeutic outcomes. Standardizing the preparation and dosing of natural products like curcumin and resveratrol is essential for achieving reliable clinical results. Bioavailability remains a primary obstacle, as many natural products are metabolized rapidly or have low solubility, which limits their efficacy in clinical settings. The emerging strategies, such as nanotechnology, offer a solution by improving drug delivery and enhancing absorption, thereby increasing the clinical potential of these natural products (Huang et al., 2019; Olajide and Sarker, 2020).

Innovative drug delivery methods, including liposomal, nanoparticle, and dendrimer systems, have improved the pharmacokinetic profiles of natural products. Curcumin and EGCG encapsulated in polymeric nanoparticles were shown to have enhanced brain penetration and sustained release. These characteristics lead to higher therapeutic efficacy in animal models and hold promise for applications in human trials (Esmaealzadeh et al., 2024; Huang et al., 2024). Similarly, resveratrol-loaded liposomes have shown improved stability and bioavailability, which are crucial for maximizing its effects in neurodegenerative diseases (Arbo et al., 2020).

The regulatory bodies pose another challenge, as the complexity of natural products often complicates the approval process. Unlike synthetic drugs with single active ingredients, natural products are typically complex mixtures with multiple bioactive components, making it difficult to meet stringent regulatory standards. However, advances in analytical techniques for isolating, characterizing, and standardizing active components are facilitating progress in this area (Andrade et al., 2023). To unlock their full potential, more robust and large-scale clinical trials are required to establish both safety and efficacy and to gain regulatory acceptance for natural products as mainstream treatments for neurodegenerative diseases.

## 9 Recent advances in nanotechnology

Recent advances in nanotechnology and the integration of natural products are redefining drug delivery systems. These developments have markedly improved therapeutic applications (Huang et al., 2024). The use of natural products in nanocarriers has addressed critical issues, such as poor solubility and low bioavailability (Huang et al., 2024). Recent innovations in nanocarrier systems, including polymeric nanoparticles and liposomes, have enhanced the stability and targeted delivery of natural products (Andrade et al., 2023). Novel formulations of curcumin and resveratrol demonstrate neuroprotective properties and substantial clinical potential (Arbo et al., 2020; Esmaealzadeh et al., 2024). The advancement in research utilizing technologies such as genomics and metabolomics facilitates new natural products discoveries, providing a deeper understanding of their mechanisms within biological systems (Catanesi et al., 2021; Rivai and Umar, 2023).

However, significant challenges remain, such as regulatory hurdles and cost-effective manufacturing, which are vital to fully utilizing the potential of nanotechnology in enhancing therapeutic applications (Huang et al., 2024). Future research trends focus on the development of synergistic therapies, which could transform treatments for neurodegenerative diseases (Rahman et al., 2021; Teleanu et al., 2022).

## 10 Integration of natural products in nanotechnology

Nanotechnology represents a promising approach to drug delivery, offering innovative solutions to enhance the therapeutic efficacy of natural products (Huang et al., 2024). The encapsulation of natural products in nanocarriers aims to overcome significant limitations. The nanoencapsulation can solve poor solubility, low bioavailability, instability, and rapid metabolism, thereby improving their pharmacokinetic and pharmacodynamic properties (Huang et al., 2024). The role of nanocarriers, such as polymeric nanoparticles and liposomes, is crucial in protecting active natural products and enhancing solubility, thereby enabling targeted delivery to specific tissues (Fakhri et al., 2021).

The encapsulation of curcumin in polymeric nanoparticles results in improved stability and bioavailability, demonstrating significant neuroprotective effects (Esmaealzadeh et al., 2024). Liposomal formulations have similarly enhanced the therapeutic efficacy of resveratrol, evident in PD models (Arbo et al., 2020). Nanocarrier-based delivery systems facilitate controlled and sustained drug release, maintaining therapeutic concentrations and reducing the frequency of dosing, thereby enhancing treatment effectiveness (Huang et al., 2024). Targeted delivery further localizes treatments to affected tissues, optimizing therapeutic outcomes and reducing off-target toxicity (Andrade et al., 2023). Despite these promising advancements, several challenges must be addressed before widespread clinical translation.

The development of safe and effective nanocarrier formulations requires extensive optimization to balance efficacy and biocompatibility. In addition, regulatory agencies demand comprehensive safety and efficacy data, which necessitates rigorous preclinical and clinical evaluations (Huang et al., 2024). Future research should focus on optimizing large-scale production techniques, ensuring long-term safety, and integrating nanotechnology with personalized medicine approaches to maximize the clinical impact of natural product-based therapies in neurodegenerative disease management.

## 11 The future outlook and limitations

The vast biodiversity of the planet offers immense potential for the discovery of therapeutic natural products (Rivai and Umar, 2023). This rich variety not only serves as a reservoir of unique bioactive chemical structures but also presents an opportunity for significant advancements in medical interventions, particularly in neurodegenerative diseases. High-throughput screening and genomics are accelerating the identification of natural products that play crucial roles in these interventions (Lomartire and Gonçalves, 2023). By utilizing these technologies, researchers can rapidly evaluate vast libraries of secondary metabolites sourced from nature, paving the way for novel treatments (Huang et al., 2024).

A comprehensive understanding of the neuroprotective mechanisms of natural products is essential for clinical application, and for that, further research is needed. Such research should aim to elucidate the interactions of natural products with various cellular pathways involved in neuroprotection (Kim et al., 2018). Another significant challenge is the issue of poor bioavailability that commonly plagues many natural products. Advanced formulation techniques, such as nanoparticle encapsulation, and liposomal delivery offer promising solutions by significantly improving solubility, stability, and blood-brain barrier permeability (Fakhri et al., 2021). Bioavailability is a critical factor for maximizing clinical efficacy, especially in the treatment of neurodegenerative diseases (Huang et al., 2024).

Moreover, combining natural products with existing therapeutic agents could yield more effective treatment options. Synergistic effects that arise from targeting different pathways involved in disease progression could not only enhance therapeutic efficacy but also reduce drug-related side effects (Rahman et al., 2021). These strategic combinations hold immense promise, highlighting the importance of continued research into combination therapies for advancing treatment effectiveness (Teleanu et al., 2022). Overall, the integration of novel technologies, improved formulation strategies, and the exploration of combination therapies presents a robust outlook for therapeutic natural products in the realm of neurodegenerative disease treatments, emphasizing the need for ongoing research and development in this vibrant field.

Future research should prioritize the development of more sophisticated *in vivo* models that better mimic the complexity of neurodegenerative diseases, as well as conducting large-scale clinical trials to validate preclinical findings. Advanced drug delivery systems, such as nanoparticles, micelles, and dendrimers, should be further explored to enhance the bioavailability and targeted delivery of natural compounds (Arbo et al., 2020). Collaborative efforts between pharmacologists, chemists, and clinicians are essential to bridge the gap between basic research and clinical application. Long-term studies evaluating the safety and efficacy of natural products in human populations are needed to address potential side effects and establish their therapeutic viability (Andrade et al., 2023).

A significant limitation in this area of research is the diversity and complexity of natural products, which makes standardization and reproducibility challenging. Variations in extraction methods, plant origins, and chemical profiles can lead to inconsistent results. While numerous studies have demonstrated promising results in vitro models, the lack of comprehensive *in vivo* and clinical studies limits the translation of these findings to practical applications. The low bioavailability of many natural compounds, such as curcumin and resveratrol, poses a significant challenge to their clinical efficacy, despite their potent preclinical effects (Esmaealzadeh et al., 2024). The variability in study designs, including differences in dosages, models, and experimental protocols, makes it difficult to compare results and draw definitive conclusions (Fernandes et al., 2021; Jiang et al., 2023).

## 12 Conclusion

Natural products offer a promising way to develop new treatments for neurodegenerative diseases. Natural products from plants, marine organisms, and fungi, provide a variety of chemical structures and biological activities. This diversity makes them a rich source of potential therapies. Preclinical studies have shown that many natural products can reduce oxidative stress, modulate inflammation, inhibit protein aggregation, and protect neuronal health in animal models.

Early-stage clinical trials have reported encouraging results, indicating that natural products can enhance cognitive and motor functions in patients with these diseases. The use of nanocarriers to deliver natural products improves their bioavailability, stability, and targeted delivery. However, several challenges must be addressed. The insurance of the quality and standardization of natural product formulations is essential. The regulatory hurdles and the requirements for large clinical trials present significant obstacles to bringing these therapies to market. Moreover, optimizing the bioavailability and dosage of these products is crucial for their effective use in patients. Future research should aim to discover new natural sources, understand how these products work, and develop innovative delivery systems to enhance their clinical potential. The partnership among researchers, industry, and regulatory bodies is crucial for overcoming these challenges and making natural product-based therapies available to patients. Thus, natural products are a valuable and largely untapped resource for treating neurodegenerative diseases. Advanced research and development hold the potential to lead to new treatments that significantly improve the quality of life for individuals affected by these challenging conditions. It is also emphasized the limitations of *in vitro* pharmacological experiments, especially in the context of polyphenolic compounds, and it is confirmed that the reproducibility and translational value of such studies remain critical challenges in natural product research. While many reported mechanisms are well described, their direct relevance to drug development remains debatable. We see them as preliminary steps in understanding bioactivity, which require further validation in more pharmacologically relevant models. To enhance the impact of such research, the integrating *in vitro* findings with robust *in vivo*, *in vitro*, and clinical studies is essential. Moreover, improving methodological rigor such as standardizing experimental conditions, ensuring pharmacological relevance of concentrations used, and employing advanced computational approaches can help address issues of reproducibility and relevance.

## Abbreviations

NDs, Neurodegenerative diseases; AD, Alzheimer’s disease; PD, Parkinson’s disease; Aβ, Amyloid-beta; APP, Amyloid precursor protein; ROS, Reactive oxygen species; RNS, Reactive nitrogen species; HD, Huntington’s disease; HTT, Huntingtin; mHTT, Mutant huntingtin protein; SIN-1, 3-Morpholinosydnonimine; EGCG, Epigallocatechin gallate; CBD, Cannabidiol; NGF, Nerve growth factor; AMPK, AMP-activated protein kinase; SOD, Superoxide dismutase; LPS, Lipopolysaccharide.
